# Cryptococcosis Presenting as Cerebrovascular Disease

**DOI:** 10.7759/cureus.19442

**Published:** 2021-11-10

**Authors:** Bedirhan Tarhan, Yusuf Mehkri, Justin De Prey, Calvin Hu, Ibrahim S Tuna, Hans Shuhaiber

**Affiliations:** 1 Pediatric Neurology, University of Florida, Gainesville, USA; 2 Neurosurgery, University of Florida, Gainesville, USA; 3 Neurology, University of Florida, Gainesville, USA; 4 Radiology, University of Florida, Gainesville, USA

**Keywords:** cerebrovascular disease, stroke, central nervous system infection, cryptococcosis, cerebral infarction

## Abstract

Infection plays a complex role in cerebrovascular disease and is believed to have both direct and indirect mechanisms on stroke pathogenesis. if not diagnosed and treated promptly, this may have devastating consequences. Management of infection-related strokes focuses on the treatment of the underlying infection with appropriate antimicrobial drugs and the prevention of medical complications. This can lead to devastating neurological deficits. We present two cases of cryptococcal meningoencephalitis that presented with an atypical cerebral infarction. A 55-year-old male with a history of unknown autoimmune disease presented with acute onset cognitive changes and no stroke-like symptoms. A 35-year-old male with no history of autoimmune disease or other existing immunodeficiency presented with breakthrough seizure a long with stroke-like symptoms. Both patients developed multiple cerebral infarcts in multiple vascular territories, with histologic and radiologic findings consistent with a central nervous system cryptococcosis. They were subsequently diagnosed with cryptococcal meningoencephalitis and started on the appropriate anti-fungal regimen with amphotericin B and flucytosine. Prior to discharge to an inpatient rehabilitation facility, both patients were notably improved and near their neurologic baseline. It is important to understand the pathogenesis of cryptococcal infection in the central nervous system because it produces a wide variety of clinico-radiographic features that can be overlooked. Clinicians should keep infection-mediated cerebral infarcts in mind, regardless of risk factors, in order to expedite antimicrobial therapy and minimize adverse events.

## Introduction

Stroke is a leading cause of death and disability worldwide. Infection plays a complex role in cerebrovascular disease and is believed to have both direct and indirect mechanisms on stroke pathogenesis. Given the complex mechanisms of infarction related to infection, it can be difficult for clinicians to appropriately attribute stroke to an underlying infection. Management of infection-related strokes focuses on treatment of the underlying infection with appropriate antimicrobial drugs and prevention of medical complications. In the appropriate clinical context, it is essential that health care providers consider infection-mediated stroke as delay in diagnosis and treatment can lead to poor outcomes with lasting residual neurologic deficits. Here, we present two cases of cryptococcal meningoencephalitis that presented with an atypical cerebral infarction. Histologic and radiologic findings provide clues with regards to underlying pathogenesis.

## Case presentation

Case 1

A 55-year-old male with a history of type 1 diabetes mellitus (T1DM) and unspecified autoimmune disease who presented with acute onset of confusion as well as concrete visual hallucinations and behavioral change. There were no reports of any headache, fever, or stroke-like symptoms. His only outpatient medications were insulin and low-dose steroids.

**Figure 1:**
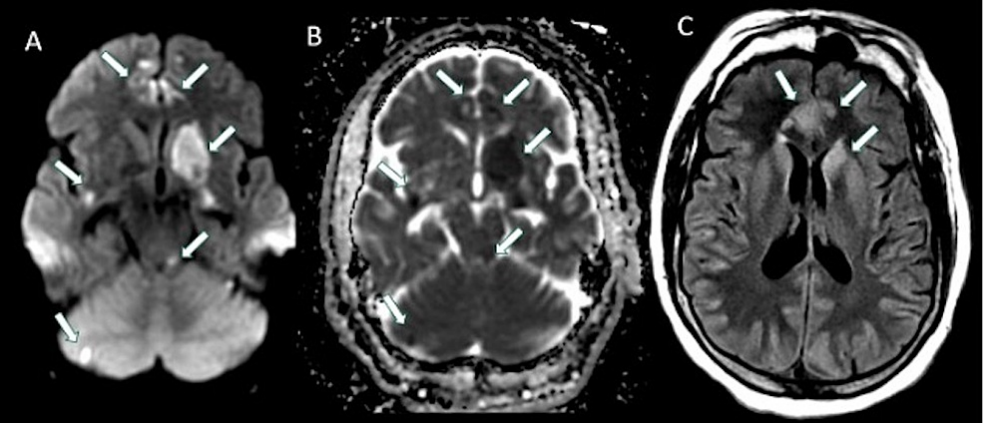
Axial DWI (A), ADC (B) and FLAIR (C) images demonstrating multifocal areas of restricted diffusion (arrows in A and B) in different vascular territories involving the left basal ganglia, bilateral medial frontal lobes, posterior insula, left superior cerebellar peduncle and right cerebellum, with corresponding increased FLAIR signal in some areas (arrows in C). DWI: diffusion-weighted imaging; ADC: apparent diffusion coefficient; FLAIR: fluid-attenuated inversion recovery.

The patient was initially admitted to an outside hospital where magnetic resonance imaging (MRI) of the brain revealed multifocal areas of restricted diffusion with areas of corresponding T2 hyperintensities on fluid-attenuated inversion recovery (FLAIR) sequences (Figure 1). There was a concern for stroke in multiple vascular territories with concern for vasculitis. Initial workup was unremarkable, and the patient was started on methylprednisolone for presumed primary central nervous system (CNS) vasculitis. He was transferred to our institution for further management by the Neurology service.

His initial neurologic exam was notable for encephalopathy, manifesting as inattention, disorientation to place and time, and stupor. He was only able to follow simple appendicular commands. Cranial nerve exam revealed left lower facial droop. He had full strength in bilateral upper extremities and 4/5 strength in bilateral lower extremities. Initial differential diagnosis included autoimmune vasculopathies, primary CNS vasculitis, and infectious meningoencephalitis given his mental status changes, reported visual hallucinations, and multifocal strokes.

**Figure 2:**
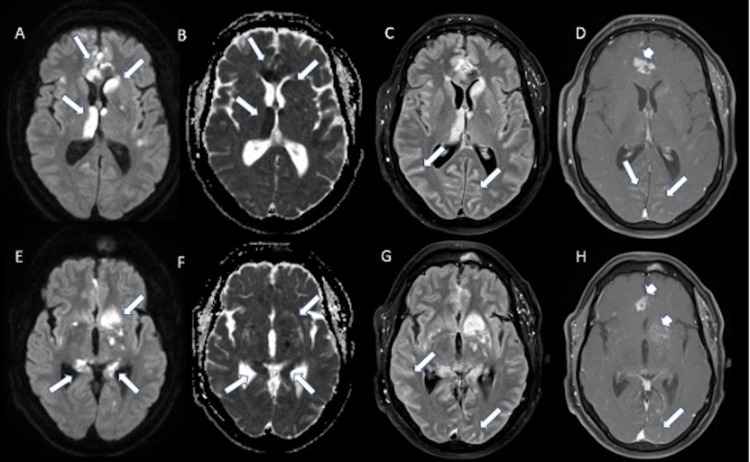
Axial DWI (A, E), ADC (B, F), fat-saturated postcontrast FLAIR (C, G) and fat-saturated T1 post-contrast (D, H) images at the level of thalamus and basal ganglia (A-D) as well as at the level of the hippocampal tail and lower basal ganglia (E-H) demonstrates multifocal areas of diffusion restriction (arrows in A, E) with corresponding ADC hypointensity (arrows in B, F) compatible with acute infarcts particularly in the medial frontal lobes, anterior body of corpus callosum, basal ganglia, medial thalamus and hippocampal tail, which are mainly along the lenticulostriate artery, ACA and PCA territories. There is diffuse incomplete suppression of CSF along the sulci on FLAIR (arrows in C, G), which also demonstrates leptomeningeal enhancement (arrows in D, H) compatible with diffuse meningitis. Areas of parenchymal enhancement in the medial frontal lobes and left basal ganglia could be due to associated meningoencephalitis as well as evolving ischemic changes (arrowheads in D, H). DWI: diffusion-weighted imaging; ADC: apparent diffusion coefficient; FLAIR: fluid-attenuated inversion recovery.

**Figure 3:**
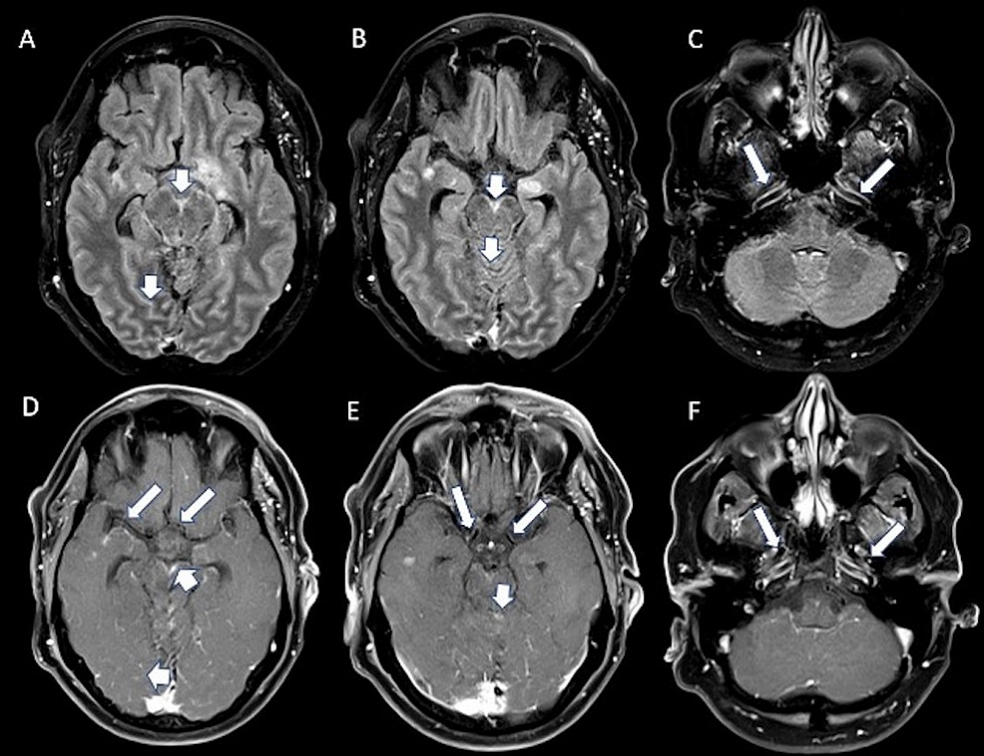
Axial postcontrast FLAIR (A-C) demonstrates diffuse incomplete suppression of CSF signal within the sulci basal cisterns particularly in the interpeduncular cistern (arrowhead in A, B), which also demonstrates mild diffuse leptomeningeal enhancement (arrowheads in D, E). There is diffuse vessel wall thickening and enhancement particularly involving the petrous carotid arteries (arrows in C, D), carotid terminus (arrows in E) as well as along the MCA and ACA (arrows in D) compatible with vessel wall inflammation. FLAIR: fluid-attenuated inversion recovery; CSF: cerebrospinal fluid; MCA: middle cerebral artery; ACA: anterior cerebral artery.

**Figure 4:**
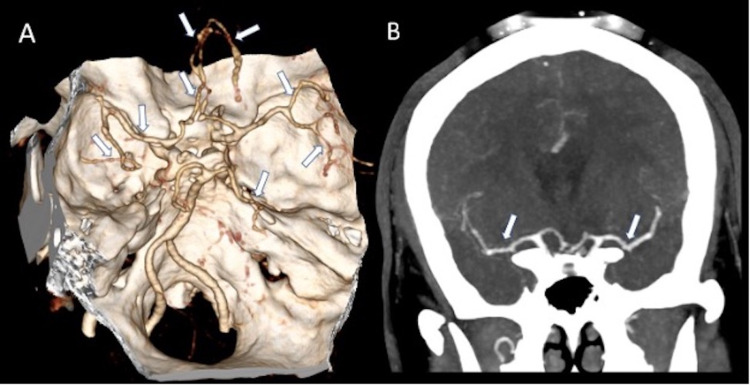
Superior view of 3D volume-rendered images from CTA (A) demonstrates diffuse vessel irregularity and multifocal areas of narrowing involving the bilateral ACA, MCA and distal PCA branches. Coronal MIP of contrast-enhanced CTA (B) demonstrates diffuse narrowing and irregularity of both MCA branches, right more than left (arrows in B). Overall findings are suggestive of meningoencephalitis associated vasculitis with inflammation, spasms, constriction, and subsequent thrombosis or necrotizing panarteritis. 3D: three dimensional; CTA: computed tomography angiography; ACA: anterior cerebral artery; MCA: middle cerebral artery; PCA: posterior cerebral artery; MIP: maximum intensity projection.

Steroids were initially held on admission to our institution until further workup could be performed. Extensive rheumatologic labs were ordered, and only rheumatoid factor and anti-CCP were found to be mildly elevated. A contrast-enhanced MRI of the brain demonstrated evolving areas of restricted diffusion with multifocal new areas of restricted diffusion in multiple vascular territories (Figure 2). There was also incomplete suppression of CSF signal on FLAIR with multiple areas of abnormal leptomeningeal enhancement, suggestive of a superimposed inflammatory process affecting the meninges (Figures 2, 3). In addition, there was abnormal vessel wall thickening and enhancement, particularly involving the intracranial carotid arteries as well as anterior cerebral arteries (ACA) and middle cerebral arteries (MCA) (Figure 3). Computed tomography angiography (CTA) of the head also demonstrated areas of vessel irregularity and multifocal areas of narrowing, particularly involving the ACA (Figure 4). On further review of the initial lab work and neuroimaging, it was felt that a primary CNS vasculitis was unlikely. Rather, findings were more suspicious for a meningoencephalitis complicated by acute stroke.

A lumbar puncture (LP) was subsequently performed, which demonstrated a lymphocytic pleocytosis (WBC 25 K/cumm, 80% lymphocytes), normal CSF glucose of 64 mg/dl, elevated protein of 104 mg/dl, and increased opening pressure of 28 cm H20. Infectious studies (including Syphilis screen, bacterial cultures, fungal cultures, HSV PCR, and flow cytometry) and inflammatory markers (including oligoclonal bands and IgG index) were unremarkable; however, India ink was performed on the CSF, which revealed a small number of encapsulated yeasts. He was subsequently diagnosed with cryptococcal meningoencephalitis and started on the appropriate anti-fungal regimen with amphotericin B and flucytosine.

He required daily LPs to ensure opening pressure remained less than 20 cm H2O. The patient was treated with four weeks of amphotericin B and flucytosine followed by eight weeks of fluconazole. Prior to discharge to an inpatient rehabilitation facility, the patient’s mental status was notably improved and near his neurologic baseline.

Case 2

**Figure 5:**
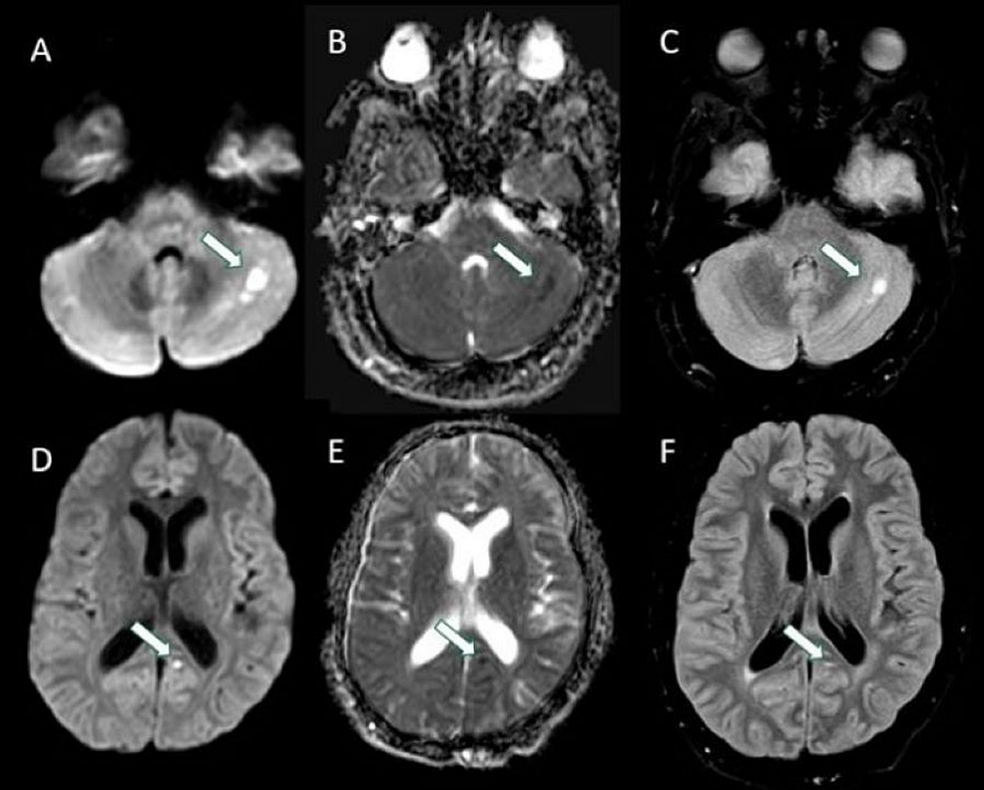
Axial DWI (A, D), ADC (B, E) and FLAIR (C, F) images demonstrating areas of restricted diffusion (arrows in A, B) in the left cerebellum and left medial occipital lobe (arrows in D, E) with corresponding increased FLAIR signal (arrows in C, F). DWI: diffusion-weighted imaging; ADC: apparent diffusion coefficient; FLAIR: fluid-attenuated inversion recovery.

A 35-year-old male with a history of hyperlipidemia and seizure disorder presented to an outside hospital following a breakthrough seizure, where he was incidentally also found to have punctate areas of acute cerebral infarcts in multiple vascular territories. Additional workup revealed the presence of a left atrial thrombus and newly diagnosed atrial fibrillation. He was ultimately discharged to home on apixaban. The patient then re-presented a month later for evaluation of transient diplopia, expressive aphasia, daily right temporal headaches, and right facial and left leg weakness. MRI of the brain showed new areas of diffusion restriction in the left cerebellar hemisphere and left medial occipital lobe (Figure 5). CTA showed no signs of carotid occlusion or stenosis. The etiology of his multifocal strokes was thought to be related to his newly diagnosed atrial fibrillation and left atrial thrombus.

**Figure 6:**
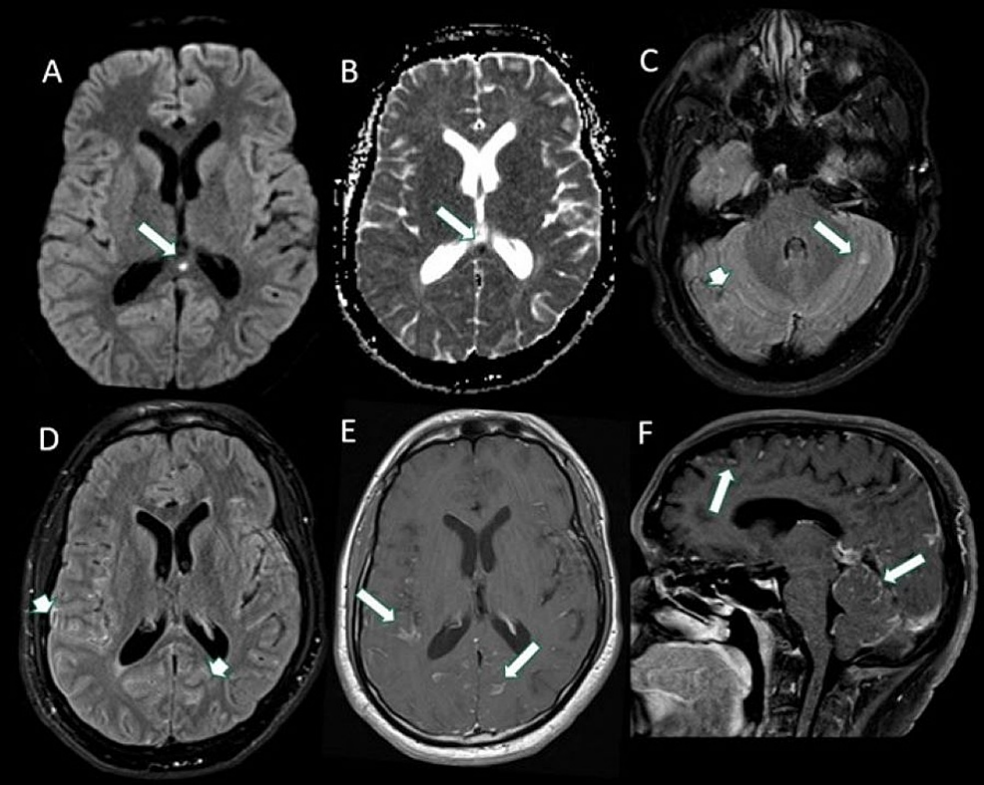
Axial DWI (A) and ADC (B) demonstrating a new area of restricted diffusion in the splenium. There is evolving FLAIR hyperintensity in the left cerebellum with multifocal areas of incomplete suppression of CSF signal on FLAIR (arrowheads in C and D). Multifocal areas of leptomeningeal enhancement (arrows in E, F) particularly in the posterior fossa. DWI: diffusion-weighted imaging; ADC: apparent diffusion coefficient; FLAIR: fluid-attenuated inversion recovery; CSF: cerebrospinal fluid.

The patient was then transferred to our hospital for further evaluation. His initial NIH stroke scale was 8 (primary deficits were including unilateral facial palsy, bilateral lower extremity pronator drift and ataxia). Stroke labs, including lipid panel and hemoglobin A1C, were unremarkable. MRI of the brain with contrast showed a new infarct in the splenium of the corpus callosum in addition to prominent generalized meningeal enhancement (Figure 6). MRI of the spine with contrast showed possible meningeal enhancement as well as punctate areas of encephalomalacia in the C3-4, C7, and T3 spinal levels. A bedside LP revealed a mildly elevated opening pressure of 24 cm H20, lymphocytic pleocytosis (WBC 150 K/cumm, 61% lymphocytes), protein 170 mg/dl, hypoglycorrhachia of 15 mg/dl, and presence of cryptococcal antigen. Other notable CSF labs included the presence of 11 oligoclonal bands. He was diagnosed with cryptococcal meningoencephalitis and started on a four-week course of amphotericin B and flucytosine. A repeat LP after several days of treatment showed a normal opening pressure of 14 cm H20, mildly improved pleocytosis (WBC 130 K/cumm, 84% lymphocytes), protein 172 mg/dl, and glucose 14 mg/dl. He did not require any additional lumbar punctures, and his symptoms (including headaches and left lower extremity weakness) gradually improved. The patient was discharged to an inpatient rehabilitation facility prior to returning home. 

Though our patient in Case 1 had a history of an unknown autoimmune disease, our patient in Case 2 had no history of autoimmune disease or other existing immunodeficiency. Both patients developed multiple cerebral infarcts in multiple vascular territories in the setting of cryptococcal meningoencephalitis, though our patient in Case 2 also had recently diagnosed with atrial fibrillation which further confounds the underlying etiology of his strokes.

## Discussion

Cryptococcosis is an infectious disease with worldwide distribution and a wide array of clinical presentations caused by pathogenic encapsulated yeasts in the genus Cryptococcus. There are only two species commonly known to cause disease in humans, including C. neoformans and C. gattii. The epidemiology of C. neoformans is well-characterized, and this organism causes disease in both immunocompromised and immunocompetent hosts [1,2].

C. neoformans disease is also likely to disseminate to the CNS. Clinical manifestations of CNS cryptococcosis include a myriad of signs and symptoms, such as headache, fever, cranial neuropathies, altered mentation, lethargy, memory loss, and signs of meningeal irritation [1-4]. Symptoms usually develop over a period of time; however, patients may occasionally present more acutely or lack typical features.

Cerebral infarction secondary to infection is a common complication, but the epidemiology of stroke in fungal infection is unreported in the literature. The clinical and radiographical features of cerebral infarction secondary to chronic fungal meningitis have been documented in case reports and case series [5-10].

CNS cryptococcosis produces a wide variety of clinico-radiographic features that may vary depending on the immunological and HIV status of a patient [8].

Cryptococcal meningoencephalitis can have normal radiographic findings in 47% of cases by CT and 8% by MRI. Approximately 21-27% of cases have typical features of cryptococcal meningoencephalitis on MRI, which are contributory but not pathognomonic [11]. These findings may include, leptomeningeal/pachymeningeal enhancement, dilated perivascular spaces, miliary nodules, plexitis, cryptococcoma, hydrocephalus, and others either in isolation or with other MRI findings [7,8,12].

It is important to understand the pathogenesis of cryptococcal infection in the CNS. Cryptococcus can spread to the CNS hematogenously and penetrate the meningeal vessels, causing meningitis. From the meninges, it can directly invade the brain parenchyma by migrating to the perivascular Virchow-Robin spaces (VRS) in addition to the subarachnoid space. Subsequent activation of inflammatory cells within the VRS leads to dilation of these spaces and deposition of inflammatory material to form mucinous pseudocysts [13]. In neuroimaging studies, this is can be represented as punctate T2 hyperintensities within the basal ganglia, thalami, midbrain, and cerebellum. These subcortical areas are predominantly supplied by the small perforating arteries of the brain. These imaging features are characteristic of cryptococcal infection of the CNS, which generally produce minimal edema and enhancement [7].

While cerebrovascular involvement is a rare entity, cerebral infarctions associated with vasculitis or other secondary complications is known to occur with cryptococcal meningoencephalitis. The presence of infarct was also found to be associated with underlying diabetes and hypertension, and there is some data to suggest higher morbidity and mortality in these patients. One study found that the incidence of cerebral infarction secondary to cryptococcal meningitis in HIV-negative patients was roughly 4% [10]. Another study, which included both HIV-positive and HIV-negative patients, found the incidence to be 13%. Interestingly, the incidence of infarction was found to be higher in patients without HIV (78%) compared to those with HIV (22%) [9]. According to the two largest published case series and numerous case reports, neurovascular involvement tends to present as multiple subcortical lacunar infarcts; however, major vascular territories or brainstem involvement may be seen on rare occasions [8-10,14-19].

There are several theories to explain the possible underlying mechanism of vascular involvement. Proposed mechanisms include, direct invasion of arterial walls leading to arteritis, venous outflow obstruction, accelerated atherosclerosis through induction of cytokines, hypercoagulability as an indirect consequence of acute infection, post-infectious infarction as a result in reduced cell-mediated immunity, among others [5,6,9,20]. Evidence of arteritis on angiography has also been observed in a patient with cryptococcal meningitis [10].

## Conclusions

In patients with atypical presentations involving cerebral infarcts, it is important to keep infection on the differential diagnosis with regards to etiology. Our cases exemplify how cryptococcal meningoencephalitis may be present regardless of risk factors and immunocompetency. Overall, clinicians should keep infection-mediated cerebral infarcts in mind to expedite antimicrobial therapy and minimize adverse events.
